# T allele at *ADIPOQ* rs1501299 G/T polymorphism is more susceptible to the influence of circulating adiponectin on arterial stiffness in nondiabetic men

**DOI:** 10.1186/s13098-018-0345-2

**Published:** 2018-06-04

**Authors:** Juhyun Song, So Ra Yoon, Oh Yoen Kim

**Affiliations:** 1 Department of Anatomy, Chonnam National University Medical School, Gwangju, 61469 South Korea; 2 Department of Food Sciences and Nutrition, Dong-A University, 37 550 beon-gil Nakdongdaero, Saha-gu, Busan, 49315 South Korea

**Keywords:** Adiponectin, Insulin resistance, Brachial-ankle pulse wave velocity, *ADIPOQ* rs1501299 G/T

## Abstract

**Background:**

Low adiponectin levels are associated with increased insulin resistance (IR) and arterial stiffness in hypertensive patients, but higher adiponectin levels are also found in heart failure patients. This discrepancy has not been fully resolved, but it may be related to the adiponectin gene (*ADIPOQ*) which regulates adiponectin production. We aimed to investigate whether the relationship between adiponectin and arterial stiffness is associated with *ADIPOQ* rs1501299 G/T polymorphism in nondiabetic Korean men.

**Methods:**

In nondiabetic men without disease (n = 301), anthropometric parameters, lipid profiles, IR, circulating adiponectin, and brachial-ankle pulse wave velocity (baPWV) were measured. rs1501299 G/T polymorphism was also analyzed.

**Results:**

Circulating adiponectin levels were negatively correlated with baPWV and homeostatic model assessment-IR in the T allele carriers (n = 167), but this correlation was not observed in the GG subjects (n = 134). However, a positive correlation between baPWV and IR was observed in the GG subjects, but not in the T carriers. These patterns were maintained after the adjustment for confounding factors. A stepwise linear regression analysis revealed that circulating adiponectin and systolic blood pressure (BP) were the main influencing factors on baPWV levels in T carriers, but systolic BP, IR and age were the main contributors to increased baPWV levels in the GG subjects.

**Conclusions:**

This study demonstrates that the relationship between circulating adiponectin and arterial stiffness is different according to *ADIPOQ* rs1501299 G/T polymorphism, and suggests that T allele is more susceptible to the influence of adiponectin on arterial stiffness than GG homozygotes. This information may prove to be useful for personal-based early prevention and management of atherosclerotic risk.

## Background

Arterial stiffness has been used as an important predictor for atherosclerosis morbidity and mortality as well as aging [1]. Accelerated arterial stiffness and vascular dysfunction have been observed in people with obesity, insulin resistance (IR), and metabolic syndrome (MetS) [2, 3]. Low levels of serum adiponectin are also associated with MetS and are considered an independent marker of peripheral arterial stiffness in hypertensive patients [4]. Adiponectin, also known as Acrp30, AdipoQ, and GBP28, is a 247-amino acid peptide that is secreted by adipocytes [5] and aortic endothelial cells [6] and circulates in large amounts (2–30 μg/mL) in plasma [7]. It has multiple functions such as insulin-sensitization, atheroprotection and cancer prevention [8, 9] by binding the adiponectin receptors (AdipoR1 and 2) on the tissues and cells [10]. Low levels of adiponectin were positively associated with IR, endothelial dysfunction, and arterial stiffness in people with metabolic disorders such as obesity, MetS, cardiovascular disease (CVD), and type 2 diabetes (T2DM) [8, 11]. However, recent studies reported that higher circulating adiponectin levels were found in heart failure patients [12] and were positively correlated with carotid intima-media thickness (CIMT) [13], thereby suggesting high adiponectin levels as a marker of CVD risk [14]. The discrepancy between these results has not been fully resolved, but we speculate that it may be related to the adiponectin gene (*ADIPOQ*, *ACDC*, or *AMP1*), a key regulator of adiponectin production and secretion [15].

Among *ADIPOQ* single nucleotide polymorphisms (SNPs), two SNPs, rs2241766 (+ 45T/G in exon 2) and rs1501299 (+ 276G/T in intron 2) have been studied mainly for their association with circulating adiponectin, obesity, IR, T2DM, and CVD risk [15, 16]. The *ADIPOQ* rs2241766 G allele has been associated with an increased incidence of T2DM [15], and the *ADIPOQ* rs1501299 T allele has been associated with decreases in IR and increases in circulating adiponectin [17]. In addition, the haplotype that combines rs2241766 T/G and rs1501299 G/T is linked to the progress of MetS and reduction of plasma adiponectin levels [16, 18]. In our previous study, *ADIPOQ* rs1501299 G/T was more strongly associated than rs2241766 T/G with several components of MetS and CVD risk, including IR, circulating triglyceride, and LDL particle size in nonobese and nondiabetic Korean men [18]. However, these associations between these two SNPs and IR are not observed in Europeans [19, 20]. This suggests that the susceptibility to IR and CVD risk is heterogeneous among ethnic groups because of different effects of *ADIPOQ* SNPs. However, it has not been investigated yet if the relationship between circulating adiponectin and arterial stiffness is associated with *ADIPOQ* SNPs, particularly in Koreans.

Therefore, this study aimed to investigate if the relationship between adiponectin and arterial stiffness is associated with *ADIPOQ* rs1501299 G/T in non-diabetic Korean men, which may provide to be useful information for early prevention and management of atherosclerotic risk.

## Methods

### Study participants

Study participants (men) were recruited from the Health Service Center in the course of a routine checkup visit or by a newspaper announcement for health examinations (January 2016–March 2016). Participants did not have any history of diseases. Exclusion criteria were the following: (1) patients diagnosed for DM and stroke; (2) any diagnosis of vascular disease, cancer (clinically or by anamnesis), renal disease, liver disease, thyroid disease, and acute or chronic inflammatory disease; (3) orthopedic limitations; and (4) ~ 10% weight loss/gain over the previous 6 months. In addition, MetS was defined using a combination and modification of the NCEP-ATPIII guidelines, Asian-Pacific guidelines, and American Diabetes Association guidelines. The definition used requires at least three of the following components: waist circumference > 90 cm (men); triglyceride ≥ 150 mg/dL; high density lipoprotein cholesterol (HDL-cholesterol) < 40 mg/dL (men); systolic/diastolic blood pressure (BP) ≥ 130/≥ 85 mmHg; and fasting glucose ≥ 100 mg/dL (fasting glucose ≥ 126 mg/mL was considered diagnostic of DM). The study participants were interviewed regarding their smoking and drinking behavior and medications. None of the participants were taking any medications (antihypertensive, antidyslipidemic, antithrombotic, and antidiabetic drugs). All the participants were provided with detailed information of the study, and participants provided written informed consent. The study protocol was approved by the Institutional Review Board of Dong-A University and was carried out in accordance with the Helsinki Declaration. Finally, 301 individuals were included in the study.

### Anthropometric parameters and blood collection

Height, body weight, and waist circumference were measured. Body mass index (BMI) was calculated as body weight divided by height in square meters (kg/m^2^). BP was obtained from seated individuals with an automatic BP monitor (HEM-7220, OMRON, Matsusaka, Japan) after a short rest. After an overnight fast, venous blood specimens were collected in EDTA-treated and untreated tubes. The tubes were immediately placed on ice until they arrived at the analytical laboratory (1–3 h). Subsequently, the blood specimens were separated into plasma or serum and stored at − 80 °C until analysis.

### Serum lipid profile

Serum total cholesterol, triglyceride, and low-density lipoprotein (LDL) were measured using commercially available kits on a Hitachi 7150 Autoanalyzer (Hitachi Ltd., Tokyo, Japan). After precipitation of serum chylomicron, LDL, and very low-density lipoprotein with dextran sulfate and magnesium chloride, HDL-cholesterol left in the supernatant was measured by an enzymatic method.

### Glucose, insulin, and HOMA-IR

Fasting glucose was measured by a glucose oxidase method (Glucose Analyzer Beckman Instruments, Irvine, CA, USA). Insulin was measured by radioimmuno-assays using commercial kits (Immuno Nucleo Corporation, Stillwater, MN, USA). IR was calculated with the homeostatic model assessment using the following equation: HOMA-IR = [fasting insulin (μIU/mL) × fasting glucose (mmol/L)]/22.5.

### Plasma adiponectin

Plasma adiponectin concentrations were measured using an enzyme immunoassay (Human Adiponectin ELISA kit, B-Bridge International Inc., San Francisco, CA, USA). The resulting color reactions were measured using the iMark™ microplate absorbance reader (Bio-Rad Laboratories, Hercules, CA, USA).

### Brachial-ankle pulse wave velocity

Brachial-ankle pulse wave velocity (baPWV) as a marker for arterial stiffness was measured using an automatic waveform analyzer (model VP-1000; Nippon Colin Ltd., Komaki, Japan). Subjects were examined in the supine position after 10 min of bed rest. Electrocardiogram electrodes were placed on both wrists, and a microphone for the phonogram was placed on the left edge of the sternum. Pneumonic cuffs were wrapped around both upper arms and ankles and connected to a plethysmographic sensor to determine the volume pulse waveform. Waveforms for the upper arm (brachial artery) and ankle (tibial artery) were stored for 10-s sample times with automatic gain analysis and quality adjustment. An oscillometric pressure sensor was attached to the cuffs to measure blood pressure at the four extremities. The baPWVs were recorded using a semiconductor pressure sensor (1200 Hz sample acquisition frequency) and calculated using the equation: (La − Lb)/ΔTba. The distance from the suprasternal notch to the elbow (Lb) or the ankle (La) were expressed by: Lb = [0.2195 × height of subject (cm)] − 2.0734, and La = [0.8129 × height of subject (cm)] + 12.328. Lb and La were defined as the distance from the aortic valve to the elbow and to the ankle, respectively. The time interval between arm and ankle distance (ΔTba) was defined as the pulse transit time between brachial and tibial arterial pressure waveforms. The average value of baPWV from both left and right sides was used in the analysis (correlation between the right and left baPWVs: r^2^ = 0.925, p < 0.0001).

### The assessment of dietary intake and physical activity level

Information on each participant’s usual diet was obtained using both a 24-h recall and a semi-quantitative food frequency questionnaire, which was previously validated [21]. We used the former to carry out analyses and the latter to check if the collected data was representative of the usual dietary pattern. All participants were given written and verbal instructions by a registered dietitian on completion of a 3-day (2 week days and 1 weekend) dietary record. Dietary energy values and nutrient content from the 3-day food records were calculated using the Computer Aided Nutritional Analysis Program (CAN-pro 4.0, Korean Nutrition Society, Seoul, Korea). Total energy expenditure (TEE) (kcal/day) was calculated from activity patterns (basal metabolic rate, 24 h-physical activity, and specific dynamic action of food).

### Genotyping of *ADIPOQ* rs1501299 G/T polymorphism

Genotyping of *ADIPOQ* rs1501299 G**/**T was performed as described previously [17, 18]. Genomic DNA was extracted from 5 mL whole blood using a commercially available DNA isolation kit (WIZARD Genomic DNA purification kit, Promega, Madison, WI, USA) according to the manufacturer’s protocol. The genotyping reaction was performed with SNP-IT assays using single primer extension technology (SNPstream 25K System, Orchid Biosystems, Princeton, NJ, USA). The DNA fragments were visualized by UV illumination using an Image Analyzer (AlphaImager 1220, Alpha Innotech, San Leandro, CA, USA). pUC19 DNA/MspI (HpaII) Marker (MBI Fermentas, Vilnius, Lithuania) was used as a control standard.

### Statistical analysis

Statistical analyses were performed with Win SPSS ver. 24.0 (Statistical Package for the Social Science, SPSS Inc., Chicago, IL, USA). Differences in continuous variables among subgroups were tested with one-way analysis of variance followed by Bonferroni correction for multiple comparisons to reduce the rate of false positives. Non-continuous variables were tested with a χ^2^ test. A general linear model analysis (GLM, post hoc multiple comparison tests) followed by Bonferroni correction was also performed with adjustment for confounding factors [i.e. age, smoking status, alcohol consumption, TEE/total calorie intake (TEE/TCI), and BMI]. Pearson and partial correlation analyses were performed for the relationship among the variables. Stepwise linear regression analysis was also used to find the major determinants for arterial stiffness. The skewed variables were log-transformed for statistical analysis. For descriptive purposes, the mean values are presented using untransformed values. Results are expressed as mean ± standard error (S.E.) or percentages. A two-tailed value of p < 0.05 was considered statistically significant.

## Results

### Clinical and biochemical parameters in study participants

Table 1 presents clinical and biochemical characteristics and dietary intake information of the study participants (n = 301). The mean age of study subjects was 47.8 ± 0.27 years (31–55 years), and the numbers (proportions) of current smokers and drinkers were 126 (41.9%) and 256 (85.5%) of all participants, respectively. The mean values of MetS related risk factors were: BMI (24.3 ± 0.14 kg/m^2^), SBP (121.1 ± 0.75 mmHg), DBP (76.3 ± 0.59 mmHg), waist circumference (86.3 ± 0.39 cm), triglyceride (136.1 ± 4.14 mg/dL), and HDL-cholesterol (52.7 ± 0.82 mg/dL). In addition, mean values of other lipid profiles and glycemic status in the study subjects were: LDL-cholesterol (113.9 ± 1.84 mg/dL), total cholesterol (193.9 ± 1.91 mg/dL), fasting glucose (91.4 ± 0.70 mg/dL), and HOMA-IR (1.74). Mean values of circulating adiponectin and baPWV were 4.81 ± 0.12 μg/mL and 1345.4 ± 8.55 mm/s, respectively. Regarding dietary intake, participants consumed an average of 2459 ± 12.6 kcal/day, with 61.9 ± 0.09% from carbohydrates, 16.8 ± 0.07% from protein and 21.3 ± 0.09% from fat. Mean values of TEE and TEE/TCI were 2377.3 ± 11.0 kcal/day and 0.97 ± 0.00, respectively.

**Table 1:** General characteristics of study subjects

Variables	Men (n = 301)	Min–max
Age (years)	47.8 ± 0.27	31.0–55.0
Current smokers (n, %)	126 (41.9)	
Current drinkers (n. %)	256 (85.0)	
Body mass index (kg/m^2^)	24.3 ± 0.14	18.5–35.0
Systolic BP (mmHg)	121.1 ± 0.75	90–170
Diastolic BP (mmHg)	76.3 ± 0.59	60–120
Waist (cm)	86.3 ± 0.39	68.5–127.0
Triglyceride (mg/dL)	136.1 ± 4.14	15–396
Total cholesterol (mg/dL)	193.9 ± 1.91	111–282
HDL-cholesterol (mg/dL)	52.7 ± 0.82	23–121
LDL-cholesterol (mg/dL)	113.9 ± 1.84	34.8–209.6
Fasting glucose (mg/dL)	91.4 ± 0.70	60–123
Insulin (μIU/mL)	7.62 ± 0.17	2.4–19.5
HOMA-IR	1.74 ± 0.04	0.4–4.4
Adiponectin (μg/mL)	4.81 ± 0.12	0.8–14.0
baPWV (mm/sec)	1345.4 ± 8.55	1002.5–1849.5
Dietary intake and energy expenditure		
TEE (kcal/day)	2377.3 ± 11.0	1749.6–2956.3
TCI (kcal/day)	2459.0 ± 12.6	1615.0–3007.0
TEE/TCI	0.97 ± 0.00	0.8–1.1
Carbohydrate (% of TCI)	61.9 ± 0.09	57.8–71.0
Protein (% of TCI)	16.8 ± 0.07	12.8–20.7
Fat (% of TCI)	21.3 ± 0.09	16.6–26.4

Results are presented as mean ± S.E. (min–max)

*BP* blood pressure; *baPWV* brachial-ankle pulse wave velocity; *HDL* high-density lipoprotein; *HOMA-IR* homeostasis model assessment of insulin resistance; *LDL* low-density lipoprotein; *TEE* total energy expenditure; *TCI* total calorie intake

### The relationships between circulating adiponectin, IR, and baPWV

**Fig. 1:**
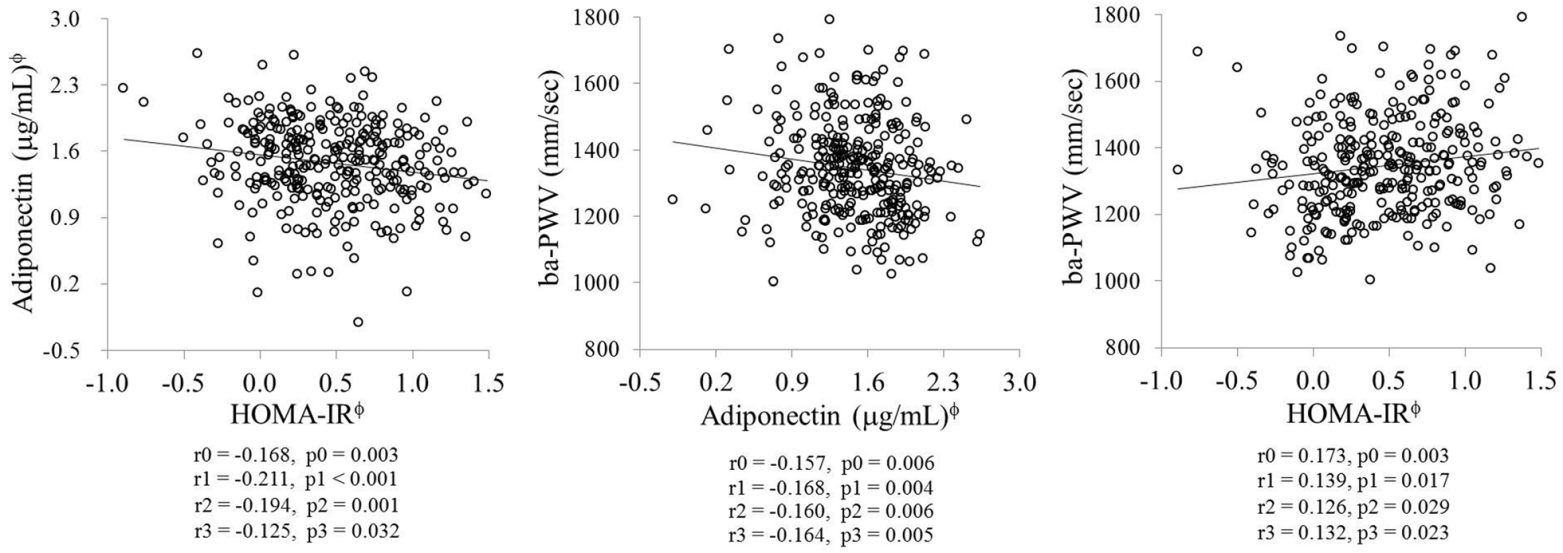
Relationships between circulating adiponectin, insulin resistance, and baPWV. r = correlation coefficient, p = *p* value; tested by *Pearson* and *partial* correlation analysis, ^ϕ^log-transformed value; r0 and p0: unadjusted; r1 and p1: adjusted for age, smoking status and alcohol consumption; r2 and p2: adjusted for age, smoking status, alcohol consumption and TEE/TCI; r3 and p3: adjusted for age, smoking status, alcohol consumption, TEE/TCI and body mass index; baPWV: brachial-ankle pulse wave velocity; HOMA-IR: homeostatic model assessment of insulin resistance; MetS: metabolic syndrome; TEE: total energy expenditure; TCI: total calorie intake

Figure 1 shows the relationships between circulating adiponectin, HOMA-IR, and baPWV. Negative correlations were observed between circulating adiponectin and HOMA-IR before and after adjustment for age, smoking status, and alcohol consumption (r0 = − 0.168, p0 = 0.003; r1 = − 0.211, p1 < 0.001). The significance of the correlations were maintained after further adjustment for TEE/TCI and BMI (r2 = − 0.194, p2 = 0.001; r3 = − 0.125, p3 = 0.032). Negative correlations were also found between circulating adiponectin and baPWV before and after adjustment for age, smoking status, and alcohol consumption (r0 = − 0.157, p0 = 0.006; r1 = − 0.168, p1 = 0.004). The significance of the correlations were maintained after further adjustment for TEE/TCI and BMI (r2 = − 0.160, p2 = 0.006; r3 = − 0.164, p3 = 0.005). However, positive correlations were observed between HOMA-IR and baPWV before and after adjustment for age, smoking status, and alcohol consumption (r0 = 0.173, p0 = 0.003; r1 = 0.139, p1 = 0.017). The significance of the correlations were maintained after further adjustment for TEE/TCI and BMI (r2 = 0.126, p2 = 0.029; r3 = 0.132, p3 = 0.023).

### Cardiometabolic risk status according to circulating adiponectin and HOMA-IR levels

**Fig. 2:**
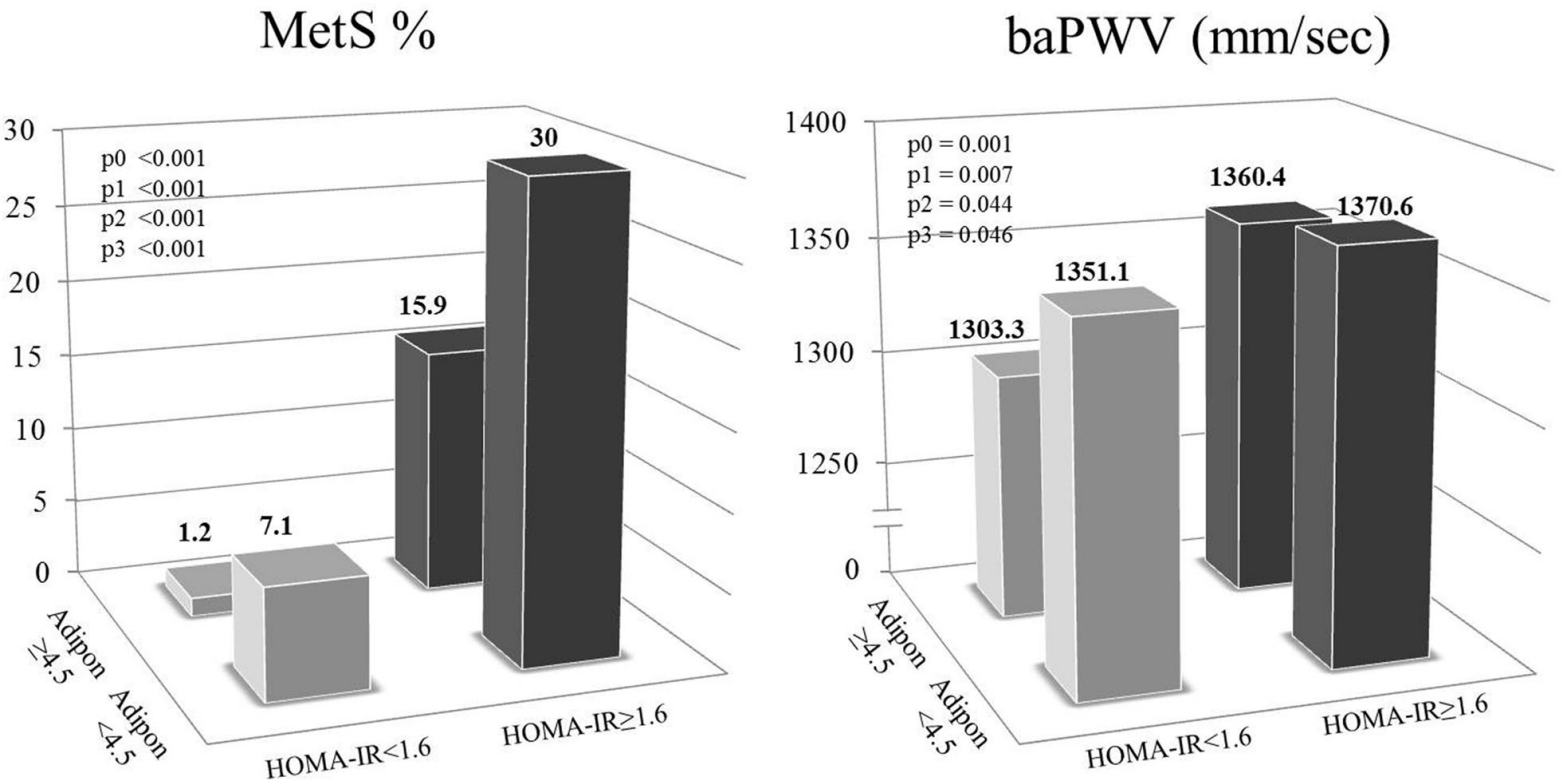
Cardiometabolic risk status according to circulating adiponectin and insulin resistance levels. Results are presented as mean ± S.E., tested by one-way analysis of variance or general linear model followed by Bonferroni method; ^ϕ^tested after log-transformed; p0: unadjusted p value; p1: p value adjusted for age, smoking status and alcohol consumption; p2: p value adjusted for age, smoking status, alcohol consumption and TEE/TCI; p3: p value adjusted for age, smoking status, alcohol consumption, TEE/TCI and body mass index; baPWV: brachial-ankle pulse wave velocity; HOMA-IR: homeostatic model assessment of insulin resistance; LDL: low-density lipoprotein; MetS: metabolic syndrome; TEE: total energy expenditure; TCI: total calorie intake

Based on the results presented in Fig. 1, study subjects were subdivided into four groups according to their median levels of circulating adiponectin and HOMA-IR: (1) higher-adiponectin (≥ 4.5)/lower-IR (< 1.6), (2) higher-adiponectin (≥ 4.5)/higher-IR (≥ 1.6), (3) lower-adiponectin (< 4.5)/lower-IR (< 1.6), and (4) lower-adiponectin (< 4.5)/higher-IR (≥ 1.6) (Fig. 2). The proportion of participants with MetS and baPWV levels were higher in the individuals with higher HOMA-IR (≥ 1.6) than those with lower HOMA-IR (< 1.6). Specifically, the proportion of participants with MetS was lowest in the higher-adiponectin (≥ 4.5)/lower-IR (< 1.6) group and highest in the lower-adiponectin (< 4.5)/higher-IR (≥ 1.6) group before and after the adjustment (p0 < 0.001, p1 < 0.001, p2 < 0.001, p3 < 0.001). Interestingly, baPWV levels were significantly lower in the individuals with higher adiponectin (≥ 4.5) than in those with lower adiponectin (< 4.5) when the HOMA-IR value was low (< 1.6) (p0 = 0.001, p1 = 0.007, p2 = 0.044, p3 = 0.046). However, baPWV levels were not significantly different between subjects with higher adiponectin (≥ 4.5) and those with lower adiponectin (< 4.5) when the HOMA-IR value was high (≥ 1.6).

### Relationships between HOMA-IR, adiponectin and baPWV in association with *ADIPOQ* rs1501299 G/T polymorphisms

**Fig. 3:**
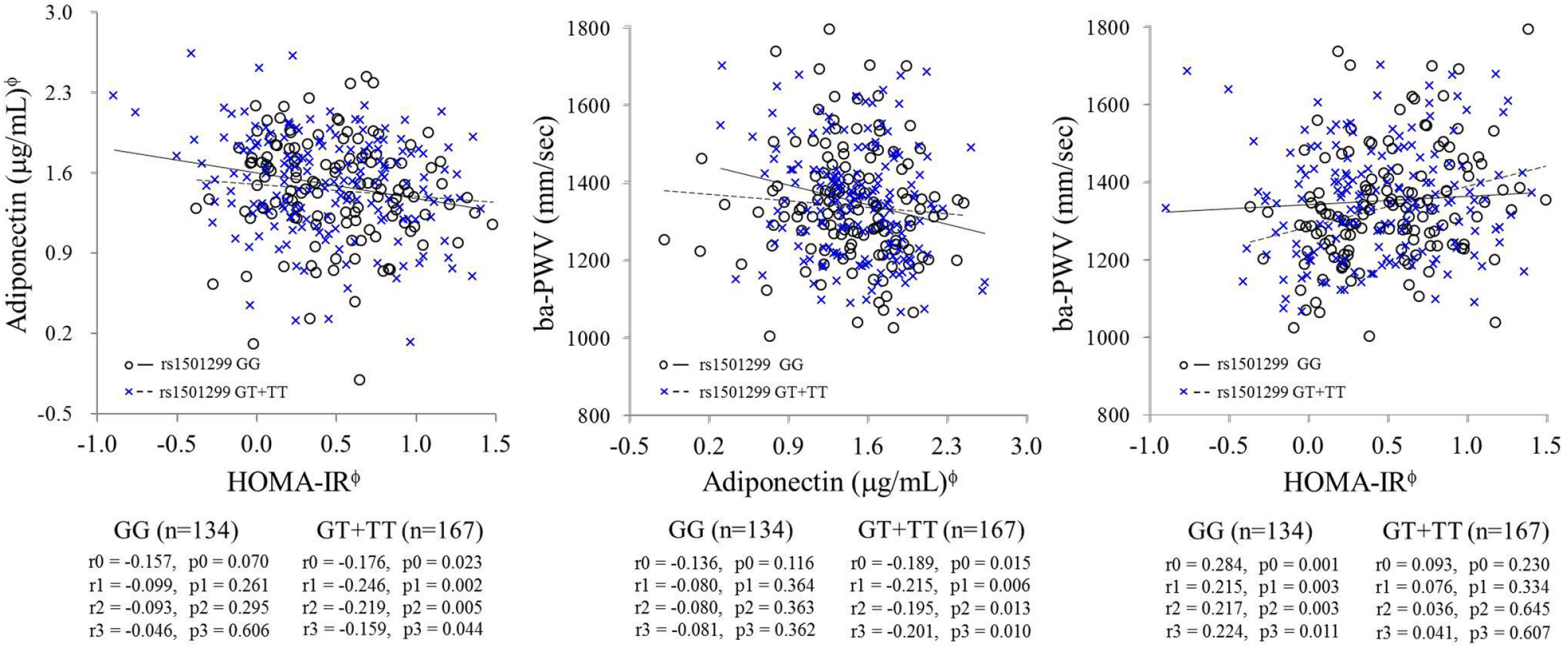
Relationships between insulin resistance, circulating adiponectin and baPWV according to *ADIPOQ* rs1501299 G > T polymorphism. r = correlation coefficient, p = p value; tested by *Pearson* and *partial* correlation analysis, ^ϕ^log-transformed value; r0 and p0: unadjusted; r1 and p1: adjusted for age, smoking status and alcohol consumption; r2 and p2: adjusted for age, smoking status, alcohol consumption and TEE/TCI; r3 and p3: adjusted for age, smoking status, alcohol consumption, TEE/TCI and body mass index; baPWV: brachial-ankle pulse wave velocity; HOMA-IR: homeostatic model assessment of insulin resistance; TEE: total energy expenditure; TCI: total calorie intake

Based on the results shown in Fig. 2, we examined if the relationships between circulating adiponectin, IR and baPWV were different according to the *ADIPOQ* rs1501299 G/T polymorphism (Fig. 3). The genotype distribution was in Hardy–Weinberg equilibrium, and the frequency of the rs1501299 T minor allele was 0.38, which was similar to that reported by previous observations in a Korean population [17, 18]. Circulating adiponectin levels were negatively correlated with HOMA-IR and baPWV in the T carriers (GT + TT, n = 167). However, these relationships were not significant in subjects with the GG genotype (n = 134). On the other hand, positive correlation between HOMA-IR and baPWV was observed in the GG subjects (n = 134), but not in the T carriers. These patterns were maintained after the adjustment for age, smoking status, alcohol consumption, TEE/TCI and BMI.

### Main contributors to baPWV levels in association with *ADIPOQ* rs1501299 G/T polymorphism

To investigate if the major contributing factors to baPWV levels were different according to *ADIPOQ* rs1501299 G/T polymorphism, we performed a stepwise linear regression analysis. We set baPWV as the dependent variable, and age, smoking status, alcohol consumption, waist circumference, systolic BP, diastolic BP, serum HDL-cholesterol, serum adiponectin level, HOMA-IR and TEE/TCI as independent variables, and then performed the analysis in total subjects, GG subjects and T carriers. As shown in Table 2, systolic BP and age were the main contributors to the increase in baPWV levels in the study subjects (β′ = 0.422, p < 0.001, β′ = 0.141, p < 0.001, respectively; and r = 0.450, p < 0.001). However, in subjects with the GG genotype, systolic BP, HOMA-IR and age were the main factors influencing baPWV levels (β′ = 0.510, p < 0.001; β′ = 0.180, p = 0.014; and β′ = 0.179, p = 0.014, respectively; r = 0.615, p = 0.014), and in the T carriers, systolic BP and serum adiponectin levels were the main factors influencing baPWV levels (β′ = 0.300, p < 0.001; and β′ = − 0.163, p = 0.029, respectively; r = 0.363, p = 0.029).

**Table 2:** Main contributors to baPWV levels in association with *ADIPOQ* rs1501299 G > T polymorphism

Study subjects	Input variables	β′	p value	*r*	*r* ^2^	p value
Total subjects	Systolic BP	0.422	< 0.001	0.450	0.203	< 0.001
	Age	0.141	< 0.001			
GG subjects	Systolic BP	0.510	< 0.001	0.615	0.379	0.014
	HOMA-IR^ϕ^	0.180	0.014			
	Age	0.179	0.014			
T carriers (GT + TT)	Systolic BP	0.300	< 0.001	0.363	0.132	0.029
	Adiponectin^ϕ^	− 0.163	0.029			

Tested by multiple stepwise regression analysis; β′: standardized beta coefficients; dependent variable: baPWV; Input variables includes age, smoking status, alcohol consumption, waist circumference, systolic BP, diastolic BP, serum HDL-cholesterol, serum adiponectin, HOMA-IR, TEE/TCI; baPWV: brachial-ankle pulse wave velocity; BP: blood pressure; HOMA-IR: homeostasis model assessment of insulin resistance; TEE: total energy expenditure; TCI: total calorie intake

^ϕ^Tested after log-transformed

## Discussion

The present study demonstrates that the relationship between circulating adiponectin and arterial stiffness expressed as baPWV is associated with the *ADIPOQ* rs1501299 G/T polymorphism in nondiabetic Korean men. Circulating adiponectin levels were negatively correlated with baPWV levels as well as HOMA-IR in rs1501299 T allele carriers, but these correlations were not significant in subjects with the GG genotype. Stepwise linear regression analysis revealed that systolic BP and circulating adiponectin were the main factors influencing baPWV levels in *ADIPOQ* rs1501299 T allele carriers. On the other hand, systolic BP, IR and age were the main contributors to increased baPWV levels in the GG subjects.

Previous studies have reported an inverse association between serum adiponectin and arterial stiffness in hypertensive patients [4, 22, 23]. According to Tsioufis et al. [23], decreased adiponectin levels lead to the elevation of mean baPWV levels in the presence of oxidative vascular injury. In a clinical study performed in 445 middle-aged Chinese participants, circulating adiponectin levels were independently and negatively associated with baPWV levels after adjustment for gender, age, BMI, number of MetS and kidney function [22]. In addition, Jia et al. [24] reported that increased arterial stiffness was directly associated with increased IR, leading to T2DM. These phenomena were also similarly observed in our subjects, but our current study additionally demonstrated that the relationships between circulating adiponectin, baPWV and IR are associated with the *ADIPOQ* rs1501299 G/T polymorphism. Several studies demonstrated that adiponectin levels could be influenced by a variant of *ADIPOQ* [15–18]. The G variant of the *ADIPOQ* rs2241766 T/G SNP is associated with decreased adiponectin levels and increased incidence of T2DM [15]. However, the minor T allele at *ADIPOQ* rs1501299 G/T polymorphism was associated with high levels of adiponectin and insulin sensitivity in non-diabetic men [17]. In addition, the *ADIPOQ* rs2241766-rs1501299 haplotype is linked to plasma adiponectin levels and the progress of MetS [16, 18]: TT/TT subjects (T/T at both rs2241766 and rs1501299) showed significantly higher adiponectin levels and lower IR than did other carriers among nonobese and nondiabetic men [18]. However, rs1501299 G/T rather than rs2241766 T/G was more strongly associated with components of MetS and CVD risk [18]. On the other hand, subjects with the T allele at rs1501299 showed significantly lower HDL-cholesterol and higher baPWV, leading to an increase in arterial stiffness in essential hypertensive patients [25]. The discrepancy among the study results may be due to the differences in study design (i.e. cross-sectional, case–control, intervention, prospective cohort, and meta-analysis studies, etc.), subject characteristics (i.e. age, sex, and ethnicity, etc.) and basic metabolic health status (i.e. super-healthy, metabolic disorders, patients with diseases, etc.) together with genetic properties. Our study subjects were all men aged between 31 and 50 years old without any history of disease or medication.

In fact, rs2241766 T/G located in exon 2 of *ADIPOQ* codes for a silent mutation, Gly15Gly (GGT to GGG) [26], which may explain why it does not strongly predispose nonobese and nondiabetic people to MetS, T2DM, or CVD compared with rs1501299 G/T [18]. Although *ADIPOQ* rs1501299 G/T is located in an intron which has had no known biological function, this SNP is associated with changes in circulating adiponectin levels, IR, and other CVD risk [15–17, 27]. Moreover, the negative associations between circulating adiponectin and HOMA-IR or baPWV were apparent in rs1501299 T allele carriers among our study subjects. Although functional genomic study is needed to elucidate the T allele effect on the metabolic relationships, we speculate that the *ADIPOQ* rs1501299 T variant alters RNA splicing or stability, leading to allele-specific differential adiponectin expression similar to the effects of intronic SNPs in the *Calpin10* and *collagen type I alpha 1 chain* genes [28, 29]. Another possible explanation is that rs1501299 is in linkage disequilibrium with other *ADIPOQ* SNPs or other genes that have biological effects on adiponectin, IR or CVD risk [25]. Furthermore, performing Mendelian randomization may affirm the effect of rs1501299 among *ADIPOQ* and other related SNPs on the relationship between circulating adiponectin and arterial stiffness. However, only rs1501299 was investigated in this study. Based on our previous and current studies, we speculate that the T allele at *ADIPOQ* rs1501299 G/T polymorphism may be an important genetic factor associated with increased adiponectin levels and decreased IR, thereby modulating arterial stiffness and lowering atherosclerotic risk in nondiabetic men.

Several studies have reported the correlation between baPWV and the onset of CVD or IR [2, 3]. Hyperinsulinemia has been known to reduce the vasodilation capacity of insulin by attenuating nitric oxide production by endothelial cells [30]. A recent study also suggested a clear relationship between IR and arterial stiffness in obese subjects [3]. In our study, a significant relationship between baPWV and HOMA-IR was observed, specifically in subjects with the GG genotype, but not in the minor T allele carriers where instead a significant association between circulating adiponectin and baPWV was observed. These phenomena may be related to the possible insufficient production of adiponectin in subjects with rs1501299 GG genotype than those with T allele [17, 18], that is, circulating adiponectin levels in the GG subjects might neither be sufficient to affect IR nor modulate arterial stiffness. Therefore, the significant association between baPWV and HOMA-IR observed in the GG subjects may be associated with other cardiometabolic risk factors besides adiponectin level. The specific mechanisms of the effect of *ADIPOQ* rs1501299 on the associations between circulating adiponectin, and IR or arterial stiffness need to be further elucidated in a larger population.

## Conclusions

The present study suggests that the T allele at *ADIPOQ* rs1501299 G/T polymorphism is more susceptible to the influence of adiponectin levels on arterial stiffness. Hence, the present results may prove to be useful for personal-based early prevention and management of atherosclerotic risk.

## Abbreviations

*ADIPOQ*: adiponectin gene
AdipoR: adiponectin receptors
baPWV: brachial-ankle pulse wave velocity
BMI: body mass index
BP: blood pressure
CAN-pro: Computer Aided Nutritional Analysis Program
CIMT: carotid intima-media thickness
CVD: cardiovascular disease
GLM: general linear model analysis
HOMA: homeostatic model assessment
HDL: high density lipoprotein cholesterol
IR: insulin resistance
MetS: metabolic syndrome
LDL: low-density lipoprotein
SNPs: single nucleotide polymorphisms
T2DM: type 2 diabetes
TCI: total calorie intake
TEE: total energy expenditure
